# Nanofibrillated Cellulose-Based Aerogels Functionalized with Tajuva (*Maclura tinctoria*) Heartwood Extract

**DOI:** 10.3390/polym13060908

**Published:** 2021-03-16

**Authors:** Rodrigo Coldebella, Marina Gentil, Camila Berger, Henrique W. Dalla Costa, Cristiane Pedrazzi, Jalel Labidi, Rafael A. Delucis, André L. Missio

**Affiliations:** 1 Laboratório de Produtos Florestais (PPGEF), Centro de Ciências Rurais, Universidade Federal de Santa Maria, 97105-900 Santa Maria, Brazil; rodrigocoldebella@yahoo.com.br (R.C.); marina_gent@hotmail.com (M.G.); camilaberger@live.com (C.B.); henriqueflorestal@ymail.com (H.W.D.C.); cpedrazzi@terra.com.br (C.P.); 2 Chemical and Environmental Engineering Department, University of the Basque Country (UPV/EHU), Plaza Europa 1, 20018 Donostia-San Sebastián, Guipuzcoa, Spain; 3 Programa de Pós-Graduação em Ciência e Engenharia de Materiais (PPGCEM), Centro de Desenvolvimento Tecnológico, Universidade Federal de Pelotas, 96010-610 Pelotas, Brazil; 4 Programa de Pós-Graduação em Ciência Ambientais (PPGCAmb), Centro de Engenharias, Universidade Federal de Pelotas, 96010-450 Pelotas, Brazil

**Keywords:** wood biorefinery, phenolic extract, wood chemistry, cellulose hydrogel

## Abstract

Aerogels are 3-D nanostructures of non-fluid colloidal interconnected porous networks consisting of loosely packed bonded particles that are expanded throughout their volume by gas and exhibit ultra-low density and high specific surface area. Cellulose-based aerogels can be obtained from hydrogels through a drying process, replacing the solvent (water) with air and keeping the pristine three-dimensional arrangement. In this work, hybrid cellulose-based aerogels were produced and their potential for use as dressings was assessed. Nanofibrilated cellulose (NFC) hydrogels were produced by a co-grinding process in a stone micronizer using a kraft cellulosic pulp and a phenolic extract from *Maclura tinctoria* (Tajuva) heartwood. NFC-based aerogels were produced by freeze followed by lyophilization, in a way that the Tajuva extract acted as a functionalizing agent. The obtained aerogels showed high porosity (ranging from 97% to 99%) and low density (ranging from 0.025 to 0.040 g·cm^−3^), as well a typical network and sheet-like structure with 100 to 300 μm pores, which yielded compressive strengths ranging from 60 to 340 kPa. The reached antibacterial and antioxidant activities, percentage of inhibitions and water uptakes suggest that the aerogels can be used as fluid absorbers. Additionally, the immobilization of the Tajuva extract indicates the potential for dentistry applications.

## 1. Introduction

One of the main technological fields in pharmaceutical research deals with the production of new multifunctional materials for the immobilization and controlled release of drugs according to the severity of each targeted injury. For Gustaite and co-workers [1], unlike traditional dressings, modern bandages must go beyond preventing wound infection, also accelerating their healing process. According to Rydzkowski and co-workers (2019) [2], personalized dressings should be effective in inhibiting bacterial growth during wound healing. 

Plant derivatives, such as (micro/nano)fibrillated cellulose (NFC), have been used in biomedical engineering due to their low toxicity, biodegradability, and biocompatibility [3,4]. NFC is a broadly flexible interconnected web-like fibril network produced through a mechanical process and composed of both crystalline and amorphous regions with length greater than 1 μm, width of 5–100 nm, and aspect ratio of 10–100 [5]. Increases in mechanical and thermal performances are the main effects attributed to the incorporation of NFC in polymeric nanocomposites [6].

According to Mokhena and John [5], non-woven absorbent webs are the second most important cellulosic product. Their main medical applications include organ repair or replacement, drug delivery, medical implants, dental fills, medical diagnostics, and instrumentation [5]. NFC-based aerogels are environmentally friendly and multi-functional materials with great potential in many facets of applications, including waste adsorption, oil/water separation, heat insulation, biomedical materials, and metal nanoparticle/metal oxide carriers [7]. Among their main features, they were reported to have a specific surface area of 10–975 m^2^·g^−1^, a porosity of 84.0–99.9%, and good biodegradability [8]. For instance, Geng [9] developed an NFC-based aerogel and reported a macroporous 3D structure with low density (0.0820–0.0083 g·cm^−3^) and high porosity (90–99%). 

These materials can be produced from NCF hydrogels using a freezing process, which is known as self-assembly, followed by sublimation of the frozen water, which is replaced by air, retaining the three-dimensional structure of the tangled NCF network [10,11,12]. Thus, the aqueous medium maintains the dispersion and colloidal individualization of NFC in water [13]. According to Wang and co-workers [14], compared to other drying processes (like CO_2_ drying), aerogels obtained from the liquid nitrogen freeze-drying may present higher shrinkage, higher compression strength, lower specific surface area, higher pore volume, and higher density.

However, this lyophilization process is very delicate and can introduce undesirable cracks or NFC agglomerates due to internal forces developed during the water desorption [15,16,17,18]. The insertion of other natural polymers can mitigate these negative effects, as well as conferring new characteristics to the aerogels, such as increased mechanical properties or different chemical functional groups.

Recent studies have reported the effects of collagen additions, such as mucoadhesives [19] and chitosan [20], as reinforcing agents, in addition to the starch used for blood absorption [21]. Xia and co-workers [22] produced cellulose pulp-based aerogels by the freeze–drying method and reported that the presence of either endogenous or exogenous lignin acted as mechanical reinforcement and conferred increased adsorption capacity of methylene blue. Alginate, for instance, has mucoadhesive properties, which may accelerate the healing process [23] and aids in the staggered release of the drug. According to Gonçalves and co-workers [23], the presence of the alginate in NFC aerogels may change their overall macro-pore structure, since it is capable of crosslinks with polysaccharides from the NFC, which may result in higher specific surface area and lower shrinkage.

To functionalize these materials, Moura and co-workers [24] inserted a propolis extract in bacterial cellulose membranes in order to produce a bio-based bandage able to accelerate the healing process. Voon and co-workers [25] studied porous cellulose spheres in order to produce a controlled release of curcumin (used as a drug model). Moreover, Andlinger and co-workers [26] produced aerogels from hydrogels by supercritical drying, which were incorporated with potato protein. The insertion of up to 10 wt% of this filler yielded increases in density and storage modulus. In this context, *Maclura tinctoria* is a wood species known as Tajuva, and its natural occurrence spreads from southeastern Brazil to northern South America. Its wood has high quality and fine finishing [27], and its fruits are extremely attractive to the local fauna. This plant also stands out in popular medicine, as local people use its leaves as dressings after tooth extractions to prevent pain and swelling [28]. This fact is corroborated by Lamounier and co-workers [29], who suggested that the Tajuva extract acts as a natural biocide against several bacteria from the oral cavity.

Two NFC-based inks were investigated by Nguyen and co-workers [30] for cartilage tissue engineering and one of them was incorporated with alginate. CaCl_2_ was used for crosslinking alginate-based structures. They observed that 3D bioprinted NFC/alginate (60/40 wt%) presented a high performance and was capable of supporting the growth and differentiation into chondrocytes of human-derived induced pluripotent stem cells. Markstedt and co-workers [31] studied alginate/NFC bio-inks using human nasoseptal chondrocytes, which showed cell viability from 73% to 86% after one and seven days of 3D culture. In the present study, hierarchically structured aerogels based on NFC, sodium alginate, and an extract from Tajuva heartwood were produced and characterized, prospecting wound dressing applications.

## 2. Materials and Methods

### 2.1. Production and Characterization of the Tajuva Extracts

Heartwood fragments from adult Tajuva trees were milled in a Willey mill (TECNAL^®^, model TE-680, Piracicaba, São Paulo, Brasil) and subjected to hot extraction using ethanol as a solvent and a Soxhlet extraction apparatus until exhaustion of the plant material. This extraction occurred in triplicate. Then, the crude ethanolic extract was concentrated in a rotary evaporator (TECNAL^®^, model TE-210, Piracicaba, São Paulo, Brasil) at 50 °C and kept in a desiccator until constant weight. The extracts were lyophilized (Terroni^®^, model Fauvel LH-2000/3, São Carlos, São Paulo, Brasil) for the total removal of solvent residues. 

Microbial strains of the following bacteria were used: *Chromobacterium violaceum* (ATCC 12472), *Streptococcus oralis* (clinical isolate), *Enterococcus faecalis* (ATCC 29212), *Staphylococcus aureus* (ATCC 29213), and *Escherichia coli* (ATCC 35218). The standard strains were grown on blood agar (Sigma-Aldrich, São Paulo, Brasil), incubated at 37 °C for 24 h, and adjusted using a spectrophotometer in a 0.5 McFarland standard.

The sensitivity tests of the extracts used in the aerogel formulations against Gram-positive and Gram-negative bacterial strains followed the broth microdilution method described by the Clinical and Laboratory Standards Institute (M07-A9 CLSI, 2012): 100 μL of BHI medium was added to each well and, subsequently, 100 μL of each compound solubilized in water was added in the first column, making serial dilutions and obtaining concentrations ranging from 25,000 to 48 μg × mL^−1^. Then, 15 μL of the standardized bacterial suspension was spectrophotometrically added in a 0.5 McFarland standard. The plate was then incubated at 37 °C for 24 h and the final reading was performed at naked eye.

### 2.2. Production and Characterization of the NFC-Based Hydrogels

**Figure 1:**
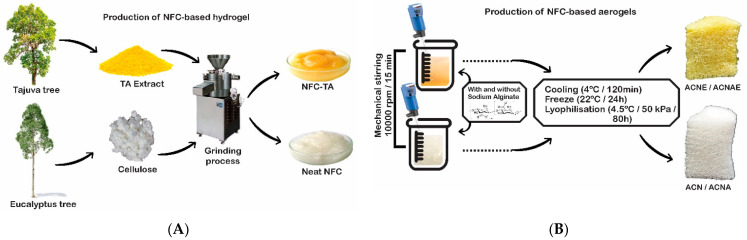
Production process schemes of the NFC-based hydrogels (**A**) and aerogels (**B**).

NFC-based hydrogels were produced following that procedure described by Missio and co-workers [32]. For that, a bleached kraft eucalyptus pulp was disaggregated and mixed with water. This suspension was inserted together with the TA extract into a Super Masscolloider Masuko Sangyo mill (Masuko^®^, model MKCA6-2J, Kawagushi, Saitama, Japan) with a rotation of 1500 rpm and 30 passes through the mill. A neat NFC-based hydrogel was also produced and the final NFC concentration was 2.2 wt% in all cases (Figure 1A).

NFC suspensions were diluted (500×) in purified water and their diameter means were accessed by Dynamic Light Scattering (DLS). The stability of the diluted suspensions was evaluated by zeta potential (ζ) values, measured with a zeta potential analyzer (Malvern Instruments^®^, model Zetasizer Nano-ZS, Worcestershire, UK). The rheological characterizations were performed using a rotational rheometer (Thermo Fisher Scientific, model Haake RheoStress1, Santa Clara, CA, USA) equipped with a standard cylinder geometry (Ø 20 mm, Z20 DIN) and a plate (Ø 35 mm, PP35). A rotational steady shear flow measurement from 1 to 100 s^−1^ and from 100 to 1 s^−1^ was performed meanwhile the samples were isothermally held at 25 °C.

### 2.3. Production and Characterization of the NFC-Based Aerogels

The NFC hydrogels were mechanically stirred (IKA Ultraturrax^®^, model T25, Staufen, Germany) at 10,000 rpm for 15 min (Figure 1B). Both neat and hybrid extract-based gels were incorporated with an aqueous solution of 2.2% sodium alginate. Each of these four mixtures was poured into cylindrical plastic containers with the dimensions of 37 × 55 mm (diameter × height) and cooled down at 4 °C for 120 min. Afterwards, the specimens were frozen at −22 °C for 24 h. The remaining moisture was removed by lyophilization (Terroni^®^, model Fauvel LH-2000/3, São Carlos, São Paulo, Brasil) at −4.5 °C and 50 kPa for 4800 min (80 h). The final aerogels were called ACN, ACNE, ACNA, and ACNAE for neat NFC, NFC + Tajuva extract, NFC + alginate, and NFC + Tajuva extract + alginate, respectively. 

FTIR-ATR spectra (Jasco^®^, model FT/IR-4100, Tokyo, Japan) were obtained in the range of 600–4000 cm^−1^ with a resolution of 4 cm^−1^ on powdered dried samples. All the FTIR spectra were normalized [0,1] using commercial software OriginPro^®^ 2017, as described by Missio and co-workers [32]. All the aerogels were measured and weighed (Shimadzu, model Aux220, Kyoto, Japan) in triplicate to determine their apparent density and average porosity. This latter parameter was calculated as (volume—mass) / (NFC density × volume). The bulk density of the NFC was 1.528 g·cm^−3^. Morphology was evaluated using scanning electron microscopy (Tescan^®^, model VEGA3, Brno, Czech Republic) at a voltage of 10 kV. Cylindrical specimens with the dimensions of 33 × 20 mm (diameter × length) were tested for compression (EMIC^®^, model DL 10000, São José dos Pinhais, Paraná, Brasil). For that, a pre-load of 0.05 N was followed by a compressive load applied at a 10 mm × min^−1^ crosshead speed until reaching 50% strain. Water uptake was evaluated by the percentage mass gain of prismatic specimens (with dimensions of 15 × 5 × 3 mm), which were immersed in distilled water until reaching constant mass. Prior to being weighted, excessive moisture of each sample was carefully removed using a filter paper. 

For the following spectroscopic analyses, the bio-based aerogels were cryofracted using liquid nitrogen and extracted in an 80% ethanol aqueous solution under mechanical stirring (Marconi, model MA562, Piracicaba, São Paulo, Brasil) for 120 min and subsequent centrifugation (Spinlab, model Spin 5000B, Ribeirão Preto, São Paulo, Brasil). The supernatant phase was removed, and the final extract was diluted in a 10% ethanol solution. Phenolic compounds and flavonoids contents, as well as antioxidant capacity, were determined in vitro by UV-VIS spectroscopy (FEMTO^®^, model 600 plus, Bosque da Saúde, São Paulo, Brasil) by means of triplicate readings of absorbance bands at 765, 510, and 517 nm, respectively. In the case of antioxidant capacity, the DPPH method was used, in which the aerogels were kept at room temperature for 24 h in total darkness. DPPH concentration was calculated using the following regression equation: Trolox concentration = 0.0045·corrected absorbance inhibition (in % − 0.0118). Gallic acid (0–80 mg·L^−1^), catechin (0–200 mg·L^−1^), and Trolox (0–0.250 mM) were used as standard solutions for preparing calibration curves to measure the contents of total phenolic, total flavonoids, and antioxidant capacity, respectively.

## 3. Results

### 3.1. TA Extracts

Table 1 shows that the aqueous heartwood extract acted against *Chromobacterium violaceum*, *Streptococcus oralis*, *Enterococcus feacalis*, and *Escherichia coli*, whereas the hydroacetic extract showed inhibitory action against all bacteria, except *Enterecoccus feacalis*. The hydroethanolic extract fought all studied bacteria. The NFC and alginate can be considered inert, since they did not show bactericidal action. Such results were similar to those published by Lamounier and co-workers [29], who investigated two Tajuva extracts (obtained from its wood and bark) and reported that these extracts were effective against six bacteria, namely *Streptococcus sanguinis*, *Streptococcus mutans*, *Streptococcus mitis*, *Prevotella nigrescens*, *Actinomyces naeslundii*, and *Porphyromonas gingivalis*. These results also indicate that the TA extract (obtained from either wood or bark) is promising for treatments in the oral cavity.

The antimicrobial activity of the TA extract is probably ascribed to its high content of phenolic compounds, such as tannins and flavonoids. In this context, the mechanism of action of phenolic compounds on pathogenic bacteria is not yet well known. According to Machado and co-workers [33], tannins and flavonoids can inactivate enzymes due to the formation of complexes with proteins and cell wall from bacteria, changing their cell membrane permeability.

### 3.2. NFC-Based Hydrogels

The hybrid hydrogel incorporated with alginate presented a slightly smaller diameter if compared to that neat NFC-based one (Table 2). According to Missio and co-workers [32], when subjected to a grinding process, the hierarchical structure of wood fibers is progressively disassembled into fibrils. Then, some new surfaces are exposed and quickly interact with surrounding water molecules, forming a strong (bound water) and thin layer. On the other hand, in a co-grinding approach, while the cellulose continually unravels, the TA extract phase may compete for bonding itself to water. Moreover, comparison of diameter means (shown in Table 1) must be restricted to the samples evaluated in the present study, since these data were obtained by DLS.

The IPD of both studied hydrogels were about 0.5, which indicates a good distribution of the NFC in the aqueous medium with neither large particles nor agglomerates [34]. In fact, IPD is the main parameter to ascertain the dispersion of nanometric particles in colloidal suspensions, depending on their molecular weight range, in which an IPD close to zero indicates a perfectly uniform particle size distribution [35]. The hybrid NFC presented a smaller zeta potential than that neat NFC hydrogel, which may be due to disruption of certain groups (like xylan and carboxyls) belonging to hemicelluloses attributed to the go-grinding in the presence of the TA extract, as aforementioned [32,36]. The studied suspensions showed negative surface charges of less than 25 mV in modulus, which indicates good stability of the suspensions [37]. Therefore, in addition to the average diameters of the NFC clusters, their stabilization and non-precipitation in aqueous media confirm the obtaining of NFC.

### 3.3. NFC-Based Aerogels

**Figure 2:**
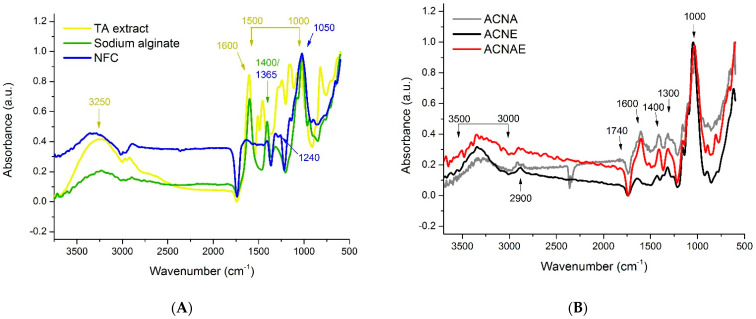
Infrared spectra of the bio-based compounds (**A**) and NFC-based hydrogels (**B**).

Figure 2A displays the infrared spectra of the TA extract, NFC, and alginate. The NFC showed its typical spectrum with remarkable bands at 700–600 cm^−1^ (ν(C–C) stretching vibration), 1050 cm^−1^ (ν(C–O) stretching vibration), 1240 and 1365 cm^−1^ (ν(C–H) bending), and 1730 cm^−1^ (ν(C=O) stretching of COOH). The TA extract showed several prominent bands in the 1000–1500 cm^−1^ range, which are attributed to C–O vibrations of (i) –C–O–C– ether bridges in complex oxygen-containing cyclic molecules and/or (ii) =C–O–C= breathing in furanic moieties, as well as in-plane C–H bending vibrations from aromatic molecules from heteroaromatic compounds [38]. That band at 1600 cm^−1^ is typically attributed to C=C stretching in the benzene ring. Finally, that broadband at 3250 cm^−1^ can be ascribed to stretching vibration of the O–H groups associated with absorbed moisture [38]. The FTIR spectra also confirmed the alginate structure as all characteristic peaks were observed at 1015, 1400, and 1600 cm^−1^ which are attributed to glycoside bond (C–O–C), symmetric stretching of –COO−, and antisymmetric stretching of –COO−, respectively [39].

Regarding the aerogels (Figure 2B), there was no clear difference between all the spectra, in which prominent bands at 1000, 1300, 1400, 1600, 1740, 2900, and 3000–3500 cm^−1^ represent –C–H stretching, aromatic skeletal vibration, –C–O vibration, –OH bending, –C=O–linkage, –CH– stretching, and OH–stretching, respectively [7].

**Figure 3:**
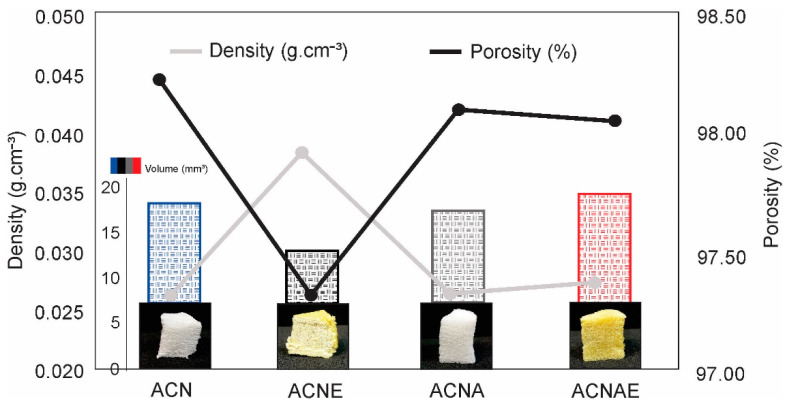
Density, porosity, and volume of the NFC-based hydrogels.

Figure 3 presents the density and porosity of all the cellulosic aerogels. The aerogels were extremely light and porous, with densities between 0.025 and 0.040 g·cm^−3^ and porosities between 97% and 99%. The aerogel density may be attributed to the density of its cellulosic raw material [40] and, because of that, that aerogel unfilled with alginate was slightly denser and bulky than the others, since it was composed by small cellulose fibrils produced through the co-grinding process, as aforementioned. The co-grinding process probably yielded a more stable cellulose hydrogel, since it destroys hydrogen bonds belonging to the NFC, and cross-linked some TA extract components, which also contributes to an increased aerogel density [41]. Therefore, the high density of the ACNE aerogel can be ascribed to its solid sheet structure and tight bonds between fibrils in the network [42].

**Figure 4:**
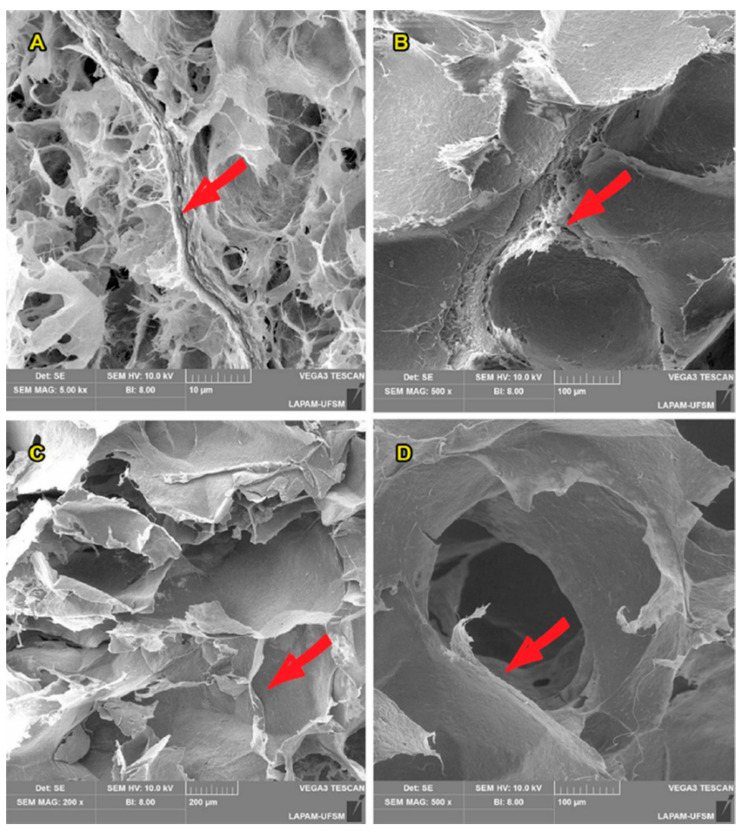
SEM micrographs of the NFC-based aerogels incorporated with alginate. Where the images (**A**,**B**) and (**C**,**D**) refer to the aerogels without and with TA extract, respectively.

The entangled NFC fibres formed a free-standing 3D structure as shown in Figure 4. The aerogels are mainly composed of 2D sheet-like membranes, which are interwoven with the NFC (Figure 4A). Additionally, the micrometre-sized pores appeared between the layered membranes. The majority of pores was from 100 to 300 μm in size, which is in agreement with some previous studies on cellulose aerogels [41,43]. Those aerogels incorporated with TA extract (shown in Figure 4C,D) presented thicker and denser cell walls compared to the neat ones (shown in Figure 4A,B), as highlighted by red arrows. 

**Figure 5:**
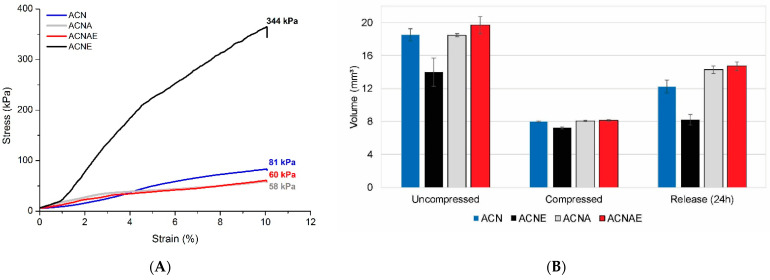
Representative stress–strain curves of the NFC-based hydrogels (**A**); Uncompressed, compressed, and release (24 h) NFC-based hydrogels volume (**B**).

All the aerogels showed ductile curves (Figure 5A), which is a favorable feature, since they can be highly compressed and retain almost the same cellular shape (as shown in Figure 5B). In addition, the compressive strength of the aerogels was found to reach approximately 60–80 kPa, although the strength of the ACNE aerogel was about five times higher than that. These mechanical properties are enough for application in wound bandages [1]. The higher compressive properties of that ACNE aerogel compared to the others can be ascribed to its higher density [42]. Additionally, these compressive features are in agreement with some already studied aerogels with similar densities [41,42,44,45].

**Figure 6:**
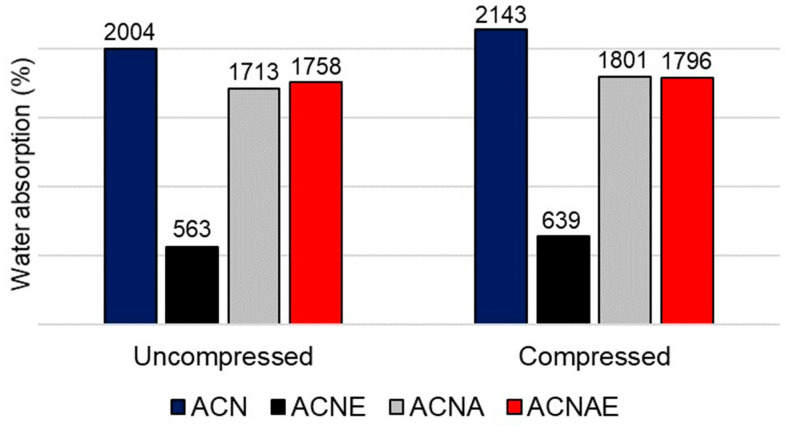
Water uptake of the NFC-based aerogels.

That stiffest aerogel (ACNE) presented the highest volume retention after the compression and the smallest water uptake (Figure 6). This behavior was also reported in literature [41,45] and can be ascribed to the aforementioned reasons. The results displayed in Figure 6 also indicate that the aerogels were capable of absorbing a large volume of water even after the compressive tests, which confirms their ductile nature and indicates that the pore structure of the aerogels was not severely damaged due to the mechanical loads. Moreover, the decreases in water uptake and apparent density accompanied the increase in compressive strength, which were attributed to the insertion of both TA extract and alginate, indirectly indicate that these additives also conferred a decrease in pore diameter and increase in a specific area, as reported in the literature [23,45]. 

The contents of total phenolic compounds and flavonoids of the aerogels and their isolated phases are presented in Table 3. The TA extract showed a high amount of both phenolic compounds and flavonoids and, because of the presence of this extract, both the ACNE and ACNAE presented significant concentrations of these compounds. These chemical properties of the TA extract overcame the same properties of some already studied extracts obtained from other plants, including *Macrosphyra Longistyla* [46], *Rhizophora racemose* [47], and *Arbutus unedo* [48]. According to Gustaite and co-workers [1], those bandages rich in polyphenols can accelerate wound healing avoiding infections and, because of that, they are promising for people who develop allergies to certain synthetic products.

The TA extract also showed a high antioxidant activity, which was also conferred to the ACNE and ACNAE aerogels in a minor intensity. This antioxidant activity can be associated with the presence of phenolic and tannin compounds [32,46]. This antioxidant activity is favorable for bandages, since it may accelerate the healing process. Additionally, this result was expected, since some previous studies already ascribed anti-HIV, antifungal, and antioxidant activities equivalent to Trolox and β-carotene to extracts from both the wood and bark from Tajuva [29]. The results obtained using the DPPH test also confirmed the presence of the TA extract in the aerogels, which yielded 78.81% inhibition for the crude extract, as well as 29.35% and 17.65% for the ACNE and ACNAE aerogels, respectively.

## 4. Conclusions

The Tajuva extract showed a high antioxidant activity probably ascribed to substances present in the extract, such as phenolic compounds, especially the tannins and other flavonoids. Enzyme inhibition assays provided indications on the potential therapeutic value of the Tajuva extract for the management of epidermal hyperpigmentation and neurodegenerative complications. However, further research including clinical in vivo studies is recommended to further evaluate these aforementioned properties, in order to include this traditional plant as a potential cosmetic, nutraceutical, and pharmacological ingredients. 

Hydrogels incorporated with NFC, sodium alginate, and TA extract were successfully produced by the cogrinding process and showed good stability, as well as a uniform particle size distribution. The aerogels with a density of 0.025–0.040 g·cm^−3^ and a porosity of 97–99% were marked by 100 to 300 μm pores distributed in a typical network and sheet-like structure. The aerogels presented good compressive strengths ranging from 60 to 340 kPa and good flexibility and durability and seem to be reusable, since they were capable of absorbing a large volume of water even after the compressive tests.

## Figures and Tables

**Table 1:** Inhibitory effects of the TA extracts.

Materials	*Chromo-bacterium violaceum* (μg)	*Staphylo-coccus aureus* (μg)	*Strepto-coccus oralis* (μg)	*Entero-coccus feacalis* (μg)	*Escheri-chia coli* (μg)
Aqueous extract	3125	na	1525	25,000	12,500
Hydroacetic extract	781	12,500	781	na	6250
Hydroethanolic extract	781	12,500	782	12,500	12,500
Alginate	na	na	na	na	na
NFC	na	na	na	na	na
NFC + alginate	na	na	na	na	na

**Table 2:** Particle diameter, polydispersity index (IPD), and zeta potential of the NFC-based hydrogels.

Hydrogels	Mean Diameter (nm)	IPD	Zeta Potential (mV)
NFC	265.3 ± 51	0.597 ± 0.11	−7.05 ± 0.9
NFC + TA extract	221.3 ± 29	0.575 ± 0.15	−11.05 ± 1.3

**Table 3:** Total phenolic and flavonoid content and antioxidant activity for the NFC-based aerogels and their isolated phases.

Treatments	Total phenolic Content	Total Flavonoid Content	DPPH (mM Trolox L^−1^)	Percentage Inhibition (%)
TA extract	43.80 ± 0.15 *	108.74 ± 3.93 *	1.74 ± 0.04 *	78.81 ± 1.91
Alginate	0.27 ± 0.02	0.45 ± 0.03	nd	nd
ACN	0.27 ± 0.02	0.60 ± 0.03	nd	nd
ACNE	4.28 ± 0.01	4.90 ± 0.05	0.13 ± 0.002	29.35 ± 0.43
ACNA	0.32 ± 0.01	0.55 ± 0.05	nd	nd
ACNAE	4.20 ± 0.01	2.81 ± 0.01	0.07 ± 0.001	17.65 ± 0.30

Where: nd= not determined. * Means ± standard deviation.

## Data Availability

The data presented in this study are available on request from the corresponding author.
